# Integrating genome and transcriptome analysis to decipher balanced structural variants in unsolved cases of neurodevelopmental disorders

**DOI:** 10.3389/fgene.2025.1603513

**Published:** 2025-07-07

**Authors:** Simona Mellone, Alice Spano, Denise Vurchio, Giulia Borgonovi, Alessandro Ugonotti, Giulia Paglino, Alba Bianco, Sara Ronzani, Maurizio Sciancalepore, Flavia Prodam, Amanda Papa, Maurizio Viri, Umberto Dianzani, Mara Giordano

**Affiliations:** ^1^ Unit of Genetics, Clinical Biochemistry, University Hospital “Maggiore della Carità”, Novara, Italy; ^2^ Department of Health Sciences, Università del Piemonte Orientale, Novara, Italy; ^3^ Division of Endocrinology, University Hospital “Maggiore della Carità”, Novara, Italy; ^4^ Department of Child Neuropsychiatry, University Hospital “Maggiore della Carità”, Novara, Italy

**Keywords:** neurodevelopmental disorders, balanced chromosomal abnormalities, *CHD7*, *SLC20A2*, *EP300*, *RBFOX3*

## Abstract

**Introduction:**

Balanced chromosomal abnormalities (BCAs) are structural variations that can underlie a wide spectrum of neurodevelopmental disorders, often remaining undetected by conventional diagnostic approaches. Whole-genome sequencing (WGS) allows for base-pair resolution of structural variants across the entire genome, making it a powerful tool to detect cryptic chromosomal rearrangements and refine breakpoint mapping. RNA sequencing (RNA-Seq), by enabling the detection of gene expression changes and fusion transcripts, provides complementary functional insights into the consequences of genomic alterations. This study integrated WGS and RNA-Seq to precisely characterize the breakpoints and assess the functional impact of *de novo* BCAs in two unsolved cases of Neurodevelopmental Disorders.

**Materials and methods:**

Short read WGS was used to identify the chromosomal breakpoints and gene disruptions caused by BCAs. RNA-Seq on blood RNA was employed to detect differential gene expression and potential fusion transcripts of disrupted genes.

**Results:**

In the first case, the inversion inv(8) (p11.2q13) disrupted two genes at the breakpoints, namely, *CHD7* and *SLC20A2*. These genes are in opposite orientations, and the inversion realigned them in the same direction, generating two novel fusion genes. Disruption of *CHD7* confirmed the suspected diagnosis of CHARGE syndrome. The interruption of *SLC20A2*, commonly associated with neurological symptoms, prompted further clinical evaluation. RNA-Seq identified in-frame fusion transcripts from the chimeric genes in the blood, suggesting a potential deleterious phenotypic effect. In the second case, WGS revealed a balanced translocation t(17; 22) (q25; q13) that disrupted *EP300* at 22q25, confirming Rubinstein-Taybi syndrome. The concurrent disruption of *RBFOX3* at 17q13 suggested additional neurological implications, particularly related to epilepsy. Transcriptomic analysis demonstrated the monoallelic and significantly reduced expression of *EP300*.

**Conclusion:**

These findings highlight the crucial role of WGS in identifying disease-associated BCAs and underscore the complementary value of RNA-Seq in assessing their functional consequences. This integrated approach enhanced diagnostic accuracy and clinical management, paving the way for more comprehensive and personalized care in these two patients.

## 1 Introduction

Balanced chromosomal abnormalities (BCAs) are structural variations in chromosomes that alter the localization or orientation of a chromosomal segment without any detectable gain or loss of genetic material. These abnormalities include translocations, inversions and complex chromosomal rearrangements (Redin et al., 2017). BCAs are relatively common, occurring in both healthy individuals and those with genetic disorders, with an estimated prevalence of one in 500 live births (Ravel et al., 2006) and are typically considered benign in the absence of overt phenotypic abnormalities. Array comparative genomic hybridization (aCGH) has demonstrated that, when clinical phenotypes are present, up to 37% of apparently balanced translocations involve cryptic copy number variations (CNVs), such as submicroscopic deletions or duplications, which can contribute to disease pathogenesis (De Gregori et al., 2007). Despite its utility in detecting large and cryptic imbalances in DNA content, aCGH cannot delineate the precise impact of chromosomal breakpoints on gene structure (Di Gregorio et al., 2017). In patients with neurodevelopmental disorders (NDDs) carrying BCAs, where aCGH fails to identify any genomic imbalances, it is hypothesized that the breakpoints themselves may exert pathogenic effects through mechanisms such as direct gene disruption, altered gene regulation due to position effects, or disturbances in parental imprinting.

Whole-genome sequencing (WGS) offers a more comprehensive approach by enabling precise breakpoint mapping, thereby uncovering subtle structural variations (SVs) that could disrupt gene function (Stranneheim et al., 2021). Gene disruption caused by SVs can manifest in multiple ways, including the truncation of protein-coding sequences, silencing of genes through deletion or displacement of regulatory elements, or the formation of pathogenic fusion transcripts due to chromosomal rearrangements (Alesi et al., 2024; Damián et al., 2021; Schluth-Bolard et al., 2019; Pagnamenta et al., 2024). Furthermore, structural variants can perturb higher-order chromatin organization within the three-dimensional (3D) genome. Chromosomal architecture is organized into topologically associating domains (TADs), which compartmentalize regulatory interactions. Translocations and inversions that disrupt TAD boundaries may lead to the mis-localization of enhancers and silencers, resulting in aberrant gene expression (Krumm and Duan, 2019). In this context, RNA sequencing (RNA-Seq) has emerged as a powerful tool in the functional interpretation of genetic variants, offering a transcriptome-wide perspective on the consequences of DNA alterations. By capturing gene expression changes, alternative splicing events, and the formation of fusion transcripts, RNA-Seq provides valuable insights into the molecular mechanisms underlying genetic disorders. Moreover, it enables the identification of allele-specific expression and regulatory disruptions that may be overlooked by DNA-based approaches. Integrating transcriptomic data with genomic findings enhances variant interpretation, facilitates the classification of pathogenic alterations, and uncovers novel disease mechanisms, making it an essential component of precision genomic medicine (Riquin et al., 2023).

To enhance our understanding of BCA effects in two patients with NDDs, we integrated WGS with RNA-seq. This combined approach not only improved the interpretation of the SVs’ impact but also demonstrated the translational potential of these methods for clinical practice, ultimately contributing to more effective and precise genomic diagnostics.

## 2 Materials and methods

### 2.1 Standard karyotype

Amniotic fluid (15–20 mL) was collected under ultrasound guidance using a sterile 20–22 G needle and processed immediately. Fetal cells were cultured in specific medium at 37°C, 5% CO_2_ for 7–10 days. Metaphase chromosomes were obtained via colchicine treatment, fixed in methanol-acetic acid (3:1). G-banding was performed using trypsin digestion and Giemsa staining. At least 20 metaphases with 400 band resolution were analyzed in each patient under a light microscope, and karyotypes were interpreted according to International System for Human Cytogenomic Nomenclature (ISCN) guidelines using an automated imaging system.

### 2.2 Fluorescence *in situ* hybridization (FISH)

Fluorescence *in situ* hybridization (FISH) was performed to investigate chromosomal rearrangements using commercially available subtelomeric probes. In Case #1, FISH was conducted on cultured amniotic fluid cells using subtelomeric probes specific for chromosome 8 (Kreatech, Leica Biosystems, Amsterdam, Netherland). The 8pter probe, labeled in green, targets the subtelomeric region of the short arm of chromosome 8 (8p23.3; locus RH65733), and the 8qter probe, labeled in red, targets the long arm subtelomeric region (8q24.3; locus D8S595). In Case #2, FISH was carried out on cultured amniotic fluid cells using subtelomeric probes for chromosomes 17q and 22q (D17S5 and D22S163, respectively; Kreatech Leica Biosystems, Amsterdam, Netherlands; distributed by Leica Biosystems, Buccinasco, Italy). Hybridization and post-hybridization washes were performed according to the manufacturer’s protocol. Fluorescent signals were visualized using a Zeiss Axio Imager.Z2 fluorescence microscope (Carl Zeiss Microscopy GmbH, Jena, Germany) and analyzed with MetaSystems Isis imaging software (MetaSystems, Altlussheim, Germany).

### 2.3 Comparative genome hybridization array

Genomic DNA was extracted from amniotic fluid using a standard purification kit and quantified via spectrophotometry. The GenetiSure Cyto 8 × 60K CGH (Agilent Technologies, Santa Clara, CA, United States) was used for high-resolution detection of copy number variations (CNVs). DNA samples (500 ng) were fluorescently labeled using the Agilent Genomic DNA Labeling Kit (Cy3 for the test sample and Cy5 for the reference), followed by purification and hybridization onto the microarray according to the manufacturer’s protocol. The slides were scanned using the Agilent SureScan Dx Microarray Scanner System, with image analysis, normalization, and annotation based on Agilent Feature Extraction Software. CNVs were identified using Agilent CytoDx Software 1.1.1.0 based on TIFF images from the data.

### 2.4 Multigene-panel sequencing

A 221 gene panel including genes correlated to NNDs was utilized (Mellone et al., 2022). DNA libraries were prepared by Sure Select QXT Target Enrichment Kit according to the protocol for Illumina Multiplex Sequencing (Agilent Technologies) as previously described (Mellone et al., 2022). Pooled samples were analyzed in parallel on MiSeq using a MiSeq sequencing reagent kit v3, 150 cycles (Illumina, Inc., San Diego, CA, United States) to obtain an estimated coverage of 150X. In each sample, the estimated coverage exceeded 50 reads for over 96% of the target gene sequence. Variant calling was executed using the enGenome eVai software (evai.engenome.com; Zucca et al., 2024).

### 2.5 Whole genome sequencing

The samples were prepared according to the Illumina TruSeq DNA sample preparation guide (Illumina) to obtain a final library of 300–400 bp average insert size. PCR was used to amplify the enriched DNA library for sequencing with a primer solution that anneals to the ends of each adapter. The BCL/cBCL (base calls) binary was converted into FASTQ using Illumina package bcl2fastq2-v2.20.0 (Illumina). Paired-end sequences were produced by NovaSeq X Instrument (Illumina) and firstly mapped to the human reference genome (GRCh38) using the mapping program Burrows-Wheeler Aligner (BWA). Based on the BAM file previously generated, variant genotyping for each sample was performed with Haplotype Caller of Genomic Analysis ToolKit (GATK-https://gatk.broadinstitute.org/hc/en-us). Variants were filtered with Variant Filtration of GATK Tool. Filtered variants were annotated with SnpEff (http://SnpEff.sourceforge.net/) and filtered with dbSNP and SNPs from the 1,000 genome project. Then, in-house program and SnpEff were used to annotate with additional databases, including: ClinVar (https://www.ncbi.nlm.nih.gov/clinvar/), dbNSFP (https://www.dbnsfp.org/) and American College of Medical Genetics and Genomics (ACMG; https://www.acmg.net/) information.

### 2.6 Breakpoint sequencing

The characterization of the identified chromosomal breakpoints and exons involved in the fusion genes was achieved through Sanger sequencing on genomic DNA. Primers were designed using Primer3 v.0.4.0 (https://primer3.ut.ee/) and the amplicon was purified with ExoSAP-IT™ Express PCR Product Cleanup Reagent (Applied Biosystems, Foster City, CA, United States). Sequencing was performed in both directions using the BigDye Terminator v1.1 Cycle Sequencing Kit (Applied Biosystems) and analyzed on a SeqStudio Genetic Analyzer (Applied Biosystems). Primer sequences are available in Supplementary Material (Supplementary Table S1).

### 2.7 RNA sequencing

Total RNA was extracted from patient peripheral blood mononuclear cells (PBMCs) isolated from whole blood using Lympholyte^®^ (Cedarlane, Burlington, NC, United States) and the miRNeasy Tissue/Cells Advanced Mini Kit (QIAGEN, Hilden, Germany). RNA concentration and purity were assessed using NanoDrop One (Thermo Fisher Scientific, Waltham, MA, United States), and RNA integrity was verified with the RNA ScreenTape Assay (Agilent Technologies) on an Agilent TapeStation 4,150, yielding an RNA integrity number.

cDNA libraries were prepared with the SureSelect XT HS2 mRNA Library Preparation System (Agilent Technologies) using 500 ng of RNA per sample. First-strand cDNA was synthesized with oligo(dT) microparticles, followed by second-strand synthesis. The cDNAs underwent end-repair, A-tailing, and ligation with molecular-barcode adaptors. After ligation, two rounds of purification were conducted, followed by targeted enrichment with specific primers. Amplification was performed, and the resulting library was cleaned up. The D1000 ScreenTape Assay in conjunction with the 4,150 TapeStation System (Agilent Technologies) assessed library quality. Finally, DNA libraries were sequenced on the NextSeq 1,000 platform (Illumina), analyzing five samples in parallel (three controls and two patients) with a NextSeq™ 1,000/2000 P2 Reagent Cartridge (300 Cycles, Illumina) that yielded over 40 million unique reads per sample. Data analysis was performed using Dynamic Read Analysis for GENomics (DRAGEN) RNA v.3.8.4 (Illumina).

### 2.8 Characterization of the fusion transcripts

Total RNA was extracted from patient-derived peripheral blood as described above. Complementary DNA (cDNA) was synthesized using the SuperScript™ III Reverse Transcriptase Kit (Thermo Fisher Scientific), following the manufacturer’s instructions. Based on breakpoint regions identified through next-generation sequencing (NGS), gene-specific primers flanking the fusion junctions of *CDH7* and *SLC20A2* were manually designed to amplify the corresponding cDNA fragments. PCR products were purified using the ExoSAP-IT™ Express PCR Product Cleanup Reagent (Applied Biosystems, Foster City, CA, United States). Bidirectional Sanger sequencing was performed using the BigDye™ Terminator v1.1 Cycle Sequencing Kit (Applied Biosystems) and analyzed on a SeqStudio™ Genetic Analyzer (Applied Biosystems). Primer sequences are listed in Supplementary Table S1.

## 3 Results

### 3.1 Case# 1

#### 3.1.1 Clinical features

Case#1 is a female, firstborn of non-consanguineous healthy parents. During the prenatal period, at 20 gestational weeks, the ultrasound showed the presence of a right aortic arch and rotation of the cerebellar vermis. Fetal magnetic resonance reported minimal asymmetry of the cerebellar hemispheres, with mild posterior rotation of the cerebellar vermis. Overall, the biometric parameters of the cerebellum were at the lower normal limits. The cisterna magna was slightly enlarged and there was thickening of the nuchal fold.

**FIGURE 1:**
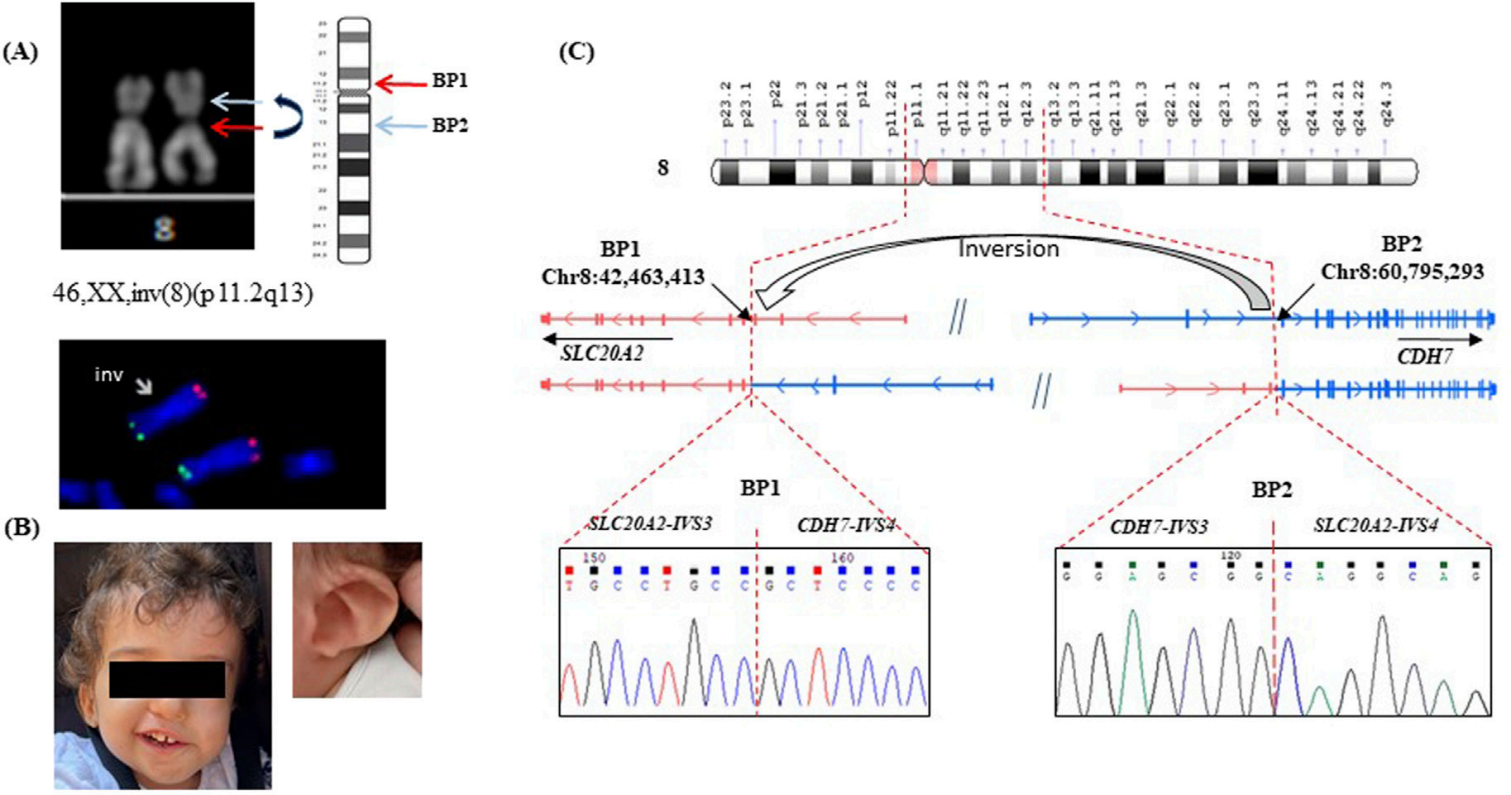
Characterization of patient #1. **(A)** karyotype 46,XX,inv(8) (p11.2q13): The arrows indicate the pericentric inversion on one copy of chromosome 8, evidenced by the altered arm lengths compared to the homologous normal chromosome. FISH confirmed the presence of a pericentric inversion involving chromosome 8, with both subtelomeric signals (8p and 8q) preserved in their expected locations: inv(8) (p11.2q13) (RH65733+, D8S595+). **(B)** patient #1 with CHARGE syndrome, a detail of the ear is shown; **(C)** Schematic representation of the pericentric inversion, indicating the positions of the two breakpoints: breakpoint 1 (BP1) on Chr8:42,463,413 within intron 3 (IVS3) of *SLC20A2* (NM_006749), and breakpoint 2 (BP2) on Chr8:60,795,293 (hg38) within intron 4 (IVS4) of *CHD7* (NM_017780). The normal *SLC20A2* and *CHD7* genes are depicted in two distinct colors, each representing the original gene sequence. The resulting chimeric genes formed by the inversion are illustrated using a combination of these colors, highlighting the fusion of sequences from both original genes. Sanger sequencing of the fused genes at each breakpoint is shown beneath the corresponding breakpoint. Genomic coordinates are based on the GRCh38 (hg38) human reference genome assembly.

Subsequent amniocentesis analysis revealed a karyotype 46,XX,inv(8) (p11.2q13) indicating a chromosomal inversion (Figure 1A) with a normal array comparative genomic hybridization (aCGH) result. The FISH analysis was carried out to further investigate the structural rearrangement identified through conventional cytogenetic analysis. The results confirmed the presence of a pericentric inversion involving chromosome 8, with both subtelomeric signals (8p and 8q) preserved in their expected locations (Figure 1A). Although the original karyotype images of the parents were not available to us, the clinical report provided by the diagnostic laboratory clearly stated that no chromosomal abnormalities were detected in either parent, indicating a *de novo* nature of the rearrangement. This was then validated at the molecular level, as detailed in the next section.

Upon birth, the patient presented with several characteristic features consistent with CHARGE syndrome (MIM #214800), including coloboma, bilateral congenital cataracts, and unilateral microphthalmia (Figure 1B). Additional findings included a seventh cranial nerve anomaly, vocal cord paralysis, and ear malformations associated with significant hearing impairment. She began assisted walking at around 24 months of age. By 3 years, she has severe hearing and visual impairments, with a height in the lower range of normal for her age. Informed consents was obtained from the patient’s parents for genetic testing.

#### 3.1.2 Genetic analysis

A targeted NGS analysis, including genes associated with neurodevelopmental disorders (NNDs) (Mellone et al., 2022), was performed. However, no pathogenic variants were identified neither in the coding regions of *CHD7*, the principal gene implicated in CHARGE syndrome, nor in any other known disease-causing genes. To determine whether the 2 breakpoints of the inversion revealed by standard karyotype fall within regulatory or intronic regions, we performed a paired-end whole-genome sequencing (WGS) with an average coverage of 30X. A pericentric inversion of 18,331,880 bp was correctly characterized by the software (Figure 1C and Supplementary Figure S1S). One breakpoint mapped at Chr8:60,795,293 (hg 38) within intron 4 of *CHD7* (NM_017780). This result was not unexpected, given that pathogenic variants in this gene are responsible for 60%–70% of CHARGE Syndrome cases (van Ravenswaaij-Arts and Martin, 2017) thus supporting the diagnosis. The second breakpoint was located at Chr8:42,463,413, within intron 3 of *SLC20A2* (NM_006749). The *SLC20A2* gene encodes a protein called sodium-dependent phosphate transporter 2 (PiT-2) that plays a significant role in phosphate homeostasis, essential for signal transduction, nucleic acid and lipid synthesis. Pathogenic variants in this gene are associated with idiopathic basal ganglia calcification (Fahr’s syndrome MIM# 213600), a condition characterized by abnormal calcium deposits in the brain (Chen et al., 2023). To substantiate the *de novo* origin of the rearrangement and compensate for the lack of direct cytogenetic images, we performed WGS also on the parent’s DNA. Analysis of the breakpoint regions revealed split reads corresponding to the rearrangement exclusively in the proband, with no such reads detected in either parent, supporting the *de novo* origin of the chromosomal inversion (Supplementary Figure S1S).

On the reference human genome (GRCh38/hg38) the two genes are arranged in opposite orientations and the here identified inversion aligns them in the same direction, leading to the creation of two novel fusion genes. Sanger sequencing of the two breakpoints did not reveal any loss/gain of genetic material at the junctions (Figure 1C).

#### 3.1.3 Transcriptome analysis

**FIGURE 2:**
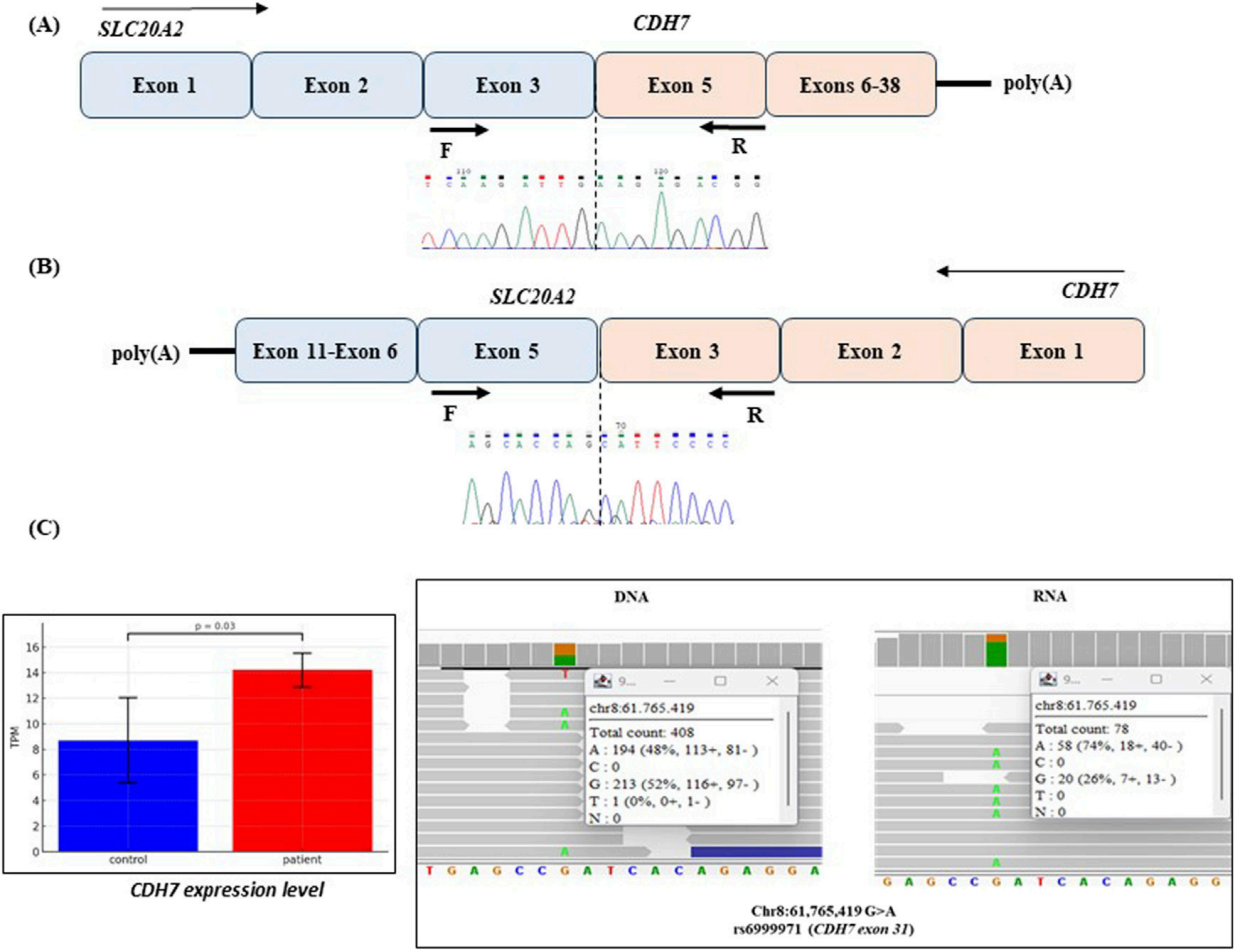
Transcriptome analysis of patient#1. **(A)** Scheme of the chimeric SLC20A2-CDH7 mRNA and Sanger sequencing of amplified blood cDNA showing the junction between exon 3 of *SLC20A2* and exon 5 of *CHD7*. **(B)** Scheme of the chimeric CDH7-SLC20A2 mRNA and Sanger sequencing showing the junction between exon 5 of *SLC20A2* and exon 3 of *CHD7*. The primers position used for amplification of cDNA are indicated for both the chimeric transcripts. **(C)** Differential allelic expression of *CHD7* RNA in the patient is quantified using TPM (Transcripts Per Million), a normalized metric accounting for gene length and sequencing depth. Allelic expression at SNP rs6999971 (c.6135G>A, exon 31 of *CHD7*) was determined by calculating the frequency of RNA-seq reads covering each allele. In genomic DNA, allele frequencies are nearly equal (A: 48%, G: 52%), whereas in RNA, expression is skewed toward the A allele (74% of total reads; *p* = 0.0169). White boxes display IGV (version 2.18.2) output at the c.6135G>A position, showing the coverage and allele percentages. Genomic coordinates correspond to the GRCh38 (hg38) human reference genome.

RNA-seq analysis was conducted on blood-RNA to detect the fusion transcripts originating from chimeric genes and to evaluate differential genes expression. A chimeric transcript combining exons 1–3 of *SLC20A2* gene with exons 5–38 of *CHD7* gene, with the skipping of exon 4 in both genes was identified. Notably, this fusion RNA remains in-frame, suggesting its potential translation. Sanger sequencing of PCR products from cDNA amplified using primers targeting exon 3 of *SLC20A2* and exon 5 of *CHD7*, confirmed the presence of the *SLC20A2-CHD7* fusion transcript (Figure 2A). Additionally, an alternatively spliced abnormal in-frame fusion transcript originating from the other side of the inversion, *CHD7-SLC20A2,* was detected joining exon 5 of *SLC20A2* and exon 3 of *CHD7* (Figure 2B) and confirmed by Sanger sequencing.

By comparing *CHD7* transcription level of the patient with 3 gender-matched healthy controls an increased expression was observed (p_adj_ = 0.03) in the patient (Figure 2C). Differential allelic expression was assessed using the SNP rs6999971 (c.6135G>A) in exon 31 of *CHD7*, that was at the heterozygous state in the patient’s DNA. The analysis revealed a skewed expression favoring the RNA bearing the A allele (Figure 2C; p = 0.0017), suggesting that the inversion alters normal gene expression level of the fused gene. This imbalance is likely attributable to an increased transcription of the fused *SLC20A2-CHD7* containing the rs6999971, as *CHD7* transcription level of is globally increased in the patient.

### 3.2 Case# 2

#### 3.2.1 Clinical features

**FIGURE 3:**
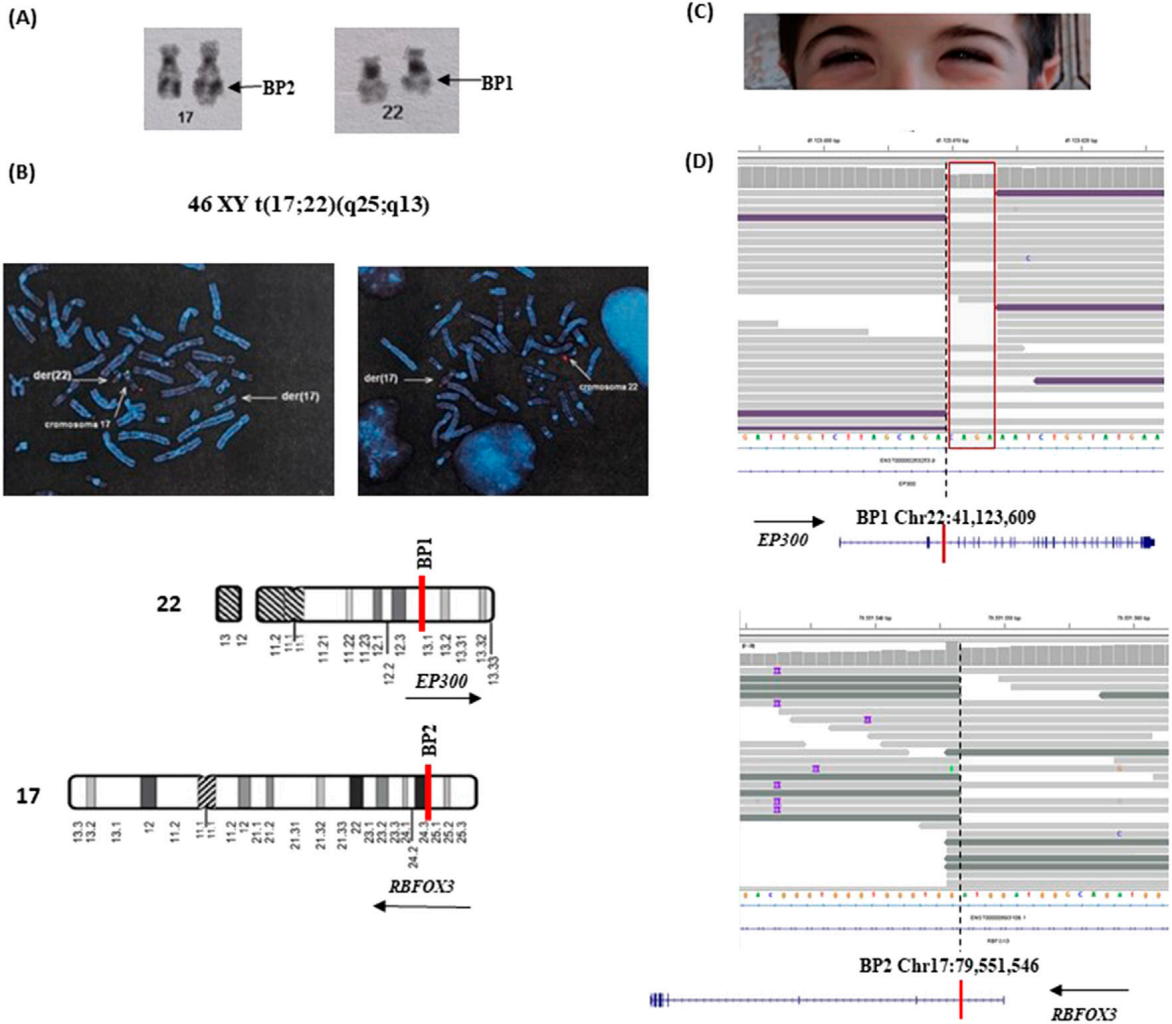
Characterization of patient#2. **(A)** Karyotype details showing the balanced translocation between chromosomes 17 and 22, designated as 46,XY,t(17; 22) (q25; q13). The derivative chromosomes are visible, with arrows indicating the breakpoints at 17q25 and 22q13. (**B**) Fluorescence in situ hybridization (FISH) analysis using subtelomeric probes. In the left panel, a green probe specific for 17p and a red probe specific for 17q highlight the translocation of the 17q region onto the derivative chromosome 22 (der(22)). In the right panel, a red probe for the 22q subtelomeric region shows its presence on the derivative chromosome 17 (der(17)), confirming the reciprocal nature of the translocation. These probe signals clearly identify the chromosomal segments involved and support the cytogenetic findings. (**C**) A detail of down-slanting palpebral fissures characteristic of Rubinstein-Taybi syndrome. **(D)** NGS read alignment at the breakpoint regions. At BP1, the loss of four nucleotides is highlighted by a drop in read coverage and marked with a red box. Below each corresponding chromosome, schematic representations depict the orientation and structure of the involved genes. Split reads span the fusion junction, indicating the precise breakpoints and confirming the translocation between the two genomic loci. Genomic coordinates refer to the GRCh38 (hg38) human reference genome assembly.

This patient is a 10-year-old boy, the first-born of healthy, non-consanguineous parents. Following a positive serum screening during pregnancy, prenatal karyotype analysis and aCGH were performed on amniotic fluid, revealing a apparently balanced translocation with karyotype 46,XY,t(17; 22) (q25; q13) (Figure 3A) and a negative aCGH result. The reciprocal translocation was confirmed through FISH with subtelomeric probes (Figure 3B) mapping on 17q and 22q, that also revealed that it arose *de novo* being absent in the parents. The parental FISH images are not available.

Prenatal ultrasounds were unremarkable, and at birth, he exhibited normal growth parameters and a ventricular septal defect, which closed spontaneously. Postnatally, he presented with short stature attributed to growth hormone deficiency correlated to a small pituitary gland, as well as a micropenis and delayed bone age. He began walking at approximately 26 months and speaking at 24 months and was diagnosed with mild global developmental delay. His characteristic features included down-slanting palpebral fissures (Figure 3C), a grimacing smile, a convex nasal profile, and dental crowding, which suggest Rubinstein-Taybi syndrome, despite the absence of hallux or thumb anomalies Based on these clinical findings, a comprehensive genetic evaluation was advised.

#### 3.2.2 Genetic analysis

To further investigate the genetic basis of the patient’s clinical phenotype, we applied a targeted next-generation sequencing (NGS) panel as previously described (Mellone et al., 2022). The bioinformatic analysis specifically focused on the *EP300* and *CREBBP* genes, which are commonly implicated in Rubinstein-Taybi syndrome (MIM #613684). However, no pathogenic variants were detected in these genes.

Subsequent whole-genome sequencing (WGS) provided a more detailed characterization of the translocation by precisely mapping the breakpoints within intron 2 of *EP300* (22q13.2) and intron one of *RBFOX3* (17q25) (Figure 3D). The translocation led to a reciprocal rearrangement, resulting in the fusion of the two genes on the two translocated chromosomes. Breakpoint analysis identified a four-nucleotide loss at the junction (Figure 3D). Also in this case WGS analysis of the patient and his parents demonstrated the presence of breakpoint-associated discordant read pairs and split reads exclusively in the proband, with no supporting evidence of such rearrangements in the parental genomes. These findings strongly indicate that both structural variants arose *de novo* (Supplementary Figure S2S). The breakpoints could not be confirmed via Sanger sequencing due to the repetitive and low-complexity nature of the region, which impairs primer design and sequencing accuracy.

*EP300* encodes a transcriptional coactivator involved in chromatin remodeling, gene expression regulation, and cellular differentiation. Pathogenic variants in *EP300* (MIM#602700), are responsible of 5%–10% of Rubinstein-Taybi syndrome (López et al., 2018). *RBFOX3* (MIM#616999), primarily expressed in neurons, regulates alternative splicing critical for neurodevelopment and synaptic plasticity. Mutations in this gene are associated with various neurological conditions, including Rolandic epilepsy, intellectual disability, autism, and alterations in sleep latency (Lal et al., 2013).

#### 3.2.3 Transcriptome analysis

**FIGURE 4:**
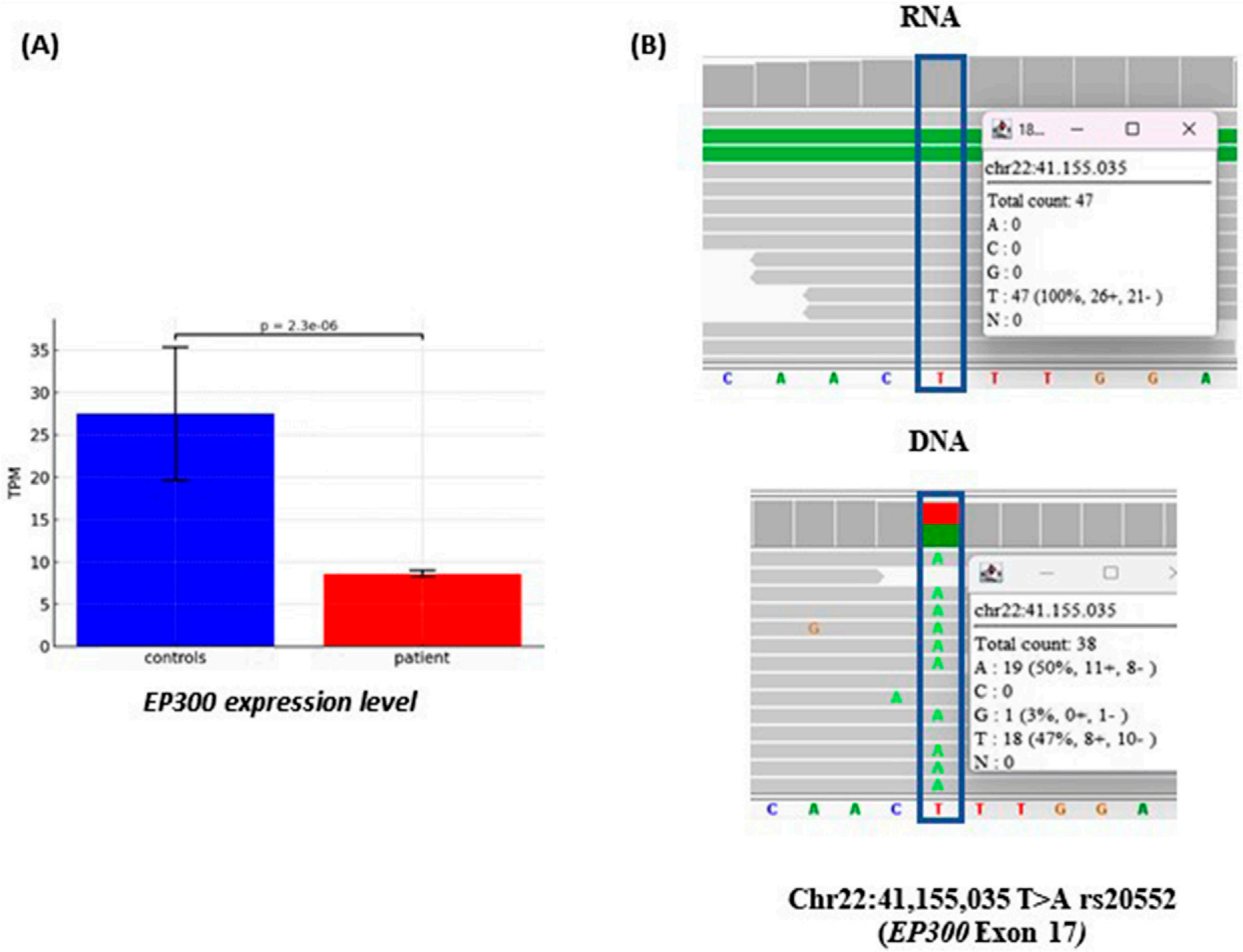
Transcriptome analysis of patient #2. (**A**) Differential allelic expression of EP300 transcripts in the patient compared to three controls, quantified using TPM (Transcripts Per Million), a normalization method that adjusts for gene length and sequencing depth. (**B**) RNAseq read alignment in patient #2 detecting the rs20552 (c.3183T>A) in exon 17 of EP300 gene. On the DNA both the alleles, A and T, are present with similar frequency, whereas in the RNA the expression is monoalleic (allele T). The single reads are randomly ranked by the software IGV; in the white squares is reported the coverage and percentage of the two alleles.

RNA Sequencing results showed a significant downregulation of *EP300* expression (p_adj_ = 2.3e^−06^) (Figure 4A) whereas the expression of *RBFOX3* could not be evaluated since it was undetectable in the blood both in the patient and in the 3 gender-matched healthy controls. As expected, no fusion transcript was detected in blood cDNA, due to the opposite transcriptional orientation of the two genes, which prevents the formation of a continuous RNA sequence. Monoallelic expression of the intact *EP300* allele was supported by RNA-level analysis of the exon 17 SNP rs20552 (c.3183T>A), which showed expression from a single allele, despite the patient being heterozygous for this variant at the genomic DNA level (Figure 4B).

## 4 Discussion

In this study, we employed WGS, complemented by RNA-seq, to investigate the functional impact of *de novo* SVs in two unsolved cases of NDDs. Our findings highlight the diagnostic utility of WGS in identifying causative genetic disruptions and uncovering unexpected candidate genes that could contribute to patients’ clinical phenotype.

In both cases, the SV disrupted two genes at the breakpoints, with at least one gene correlating with the clinical phenotype. In Case #1, disruption of *CHD7* confirmed the diagnosis of CHARGE syndrome in a patient with characteristic clinical features. However, breakpoint mapping revealed disruption also of *SLC20A2*, a gene implicated in primary familial brain calcification (PFBC, Fahr syndrome MIM# 213600). PFBC is known to manifest with a range of neurological symptoms, including movement disorders, cognitive decline, psychiatric disturbances, and epilepsy. In light of this association, the involvement of *SLC20A2* prompted further clinical investigation to assess the presence of basal ganglia calcifications or other early signs of PFBC-related pathology, guiding more comprehensive patient management and surveillance.

Similarly, in Case #2, disruption of *EP300* substantiated the suspected diagnosis of Rubinstein-Taybi syndrome. However, the concurrent disruption of *RBFOX3* at the other breakpoint on chromosome 17q25 suggests a broader impact on the patient’s neurological profile. *RBFOX3* encodes a neuronal-specific RNA-binding protein involved in alternative splicing and has been linked to epilepsy and other neurodevelopmental conditions (Lal et al., 2013). This finding emphasizes the need for continued clinical monitoring, including regular electroencephalograms (EEGs), potential adjustments to anti-seizure medications, and targeted interventions such as speech and occupational therapy.

Transcriptomic analysis further elucidated the functional consequences of these structural variants. In Case #1, we identified two in-frame fusion transcripts in the patient’s blood RNA resulting from the chromosomal inversion. The mRNA sequencing analysis revealed that the two chimeric transcripts, resulting from skipping of the exons 4 of both the genes (Figure 2A,B), prevented nonsense-mediated decay (NMD), suggesting the potential translation of a chimeric protein. The presence of the heterozygous SNP rs6999971 in exon 31 of the CHD7 gene (Figure 2C) provided a unique molecular marker to assess allele-specific expression. RNA sequencing revealed a significant overrepresentation of transcripts bearing the A allele, indicating increased expression of the chimeric mRNA composed of SLC20A2 exons 1–3 fused to CHD7 exons 5–38. This finding suggests that the chimeric transcript is not only produced but is also highly expressed.

Although the functional consequences of this fusion are currently unknown, its potential pathogenicity cannot be ruled out. If translated, the resulting fusion protein may interfere with normal cellular processes through dominant-negative or gain-of-function mechanisms. While the patient does not currently exhibit additional clinical symptoms beyond those already described, the presence of such a transcript raises the possibility of future, unanticipated phenotypic effects.

Gene fusions are well-documented in oncogenesis (Sorokin et al., 2022), but growing evidence indicates they may also play a role in neurodevelopmental disorders (NDDs). Previous studies have reported the presence of chimeric transcripts in conditions such as schizophrenia (Rippey et al., 2013) and autism spectrum disorder (Bacchelli et al., 2019), underscoring the need for further functional investigation into their contribution to NDD pathogenesis.

Current diagnostic strategies for NDDs primarily rely on exome sequencing (ES) or targeted gene panels, often supplemented by copy number variation (CNV) analysis. While effective for detecting single-nucleotide variants (SNVs), small insertions/deletions, and unbalanced rearrangements, these approaches are insufficient for identifying balanced rearrangements, deep intronic variants, or complex SVs. The present study demonstrates the added value of WGS in delineating structural rearrangements at nucleotide resolution, leading to the identification of additional genes, namely, *SLC20A2* and *RBFOX3*, not initially suspected based on the patients’ clinical presentation. This underscores the importance of integrating WGS into clinical workflows for unresolved NDD cases, particularly those with balanced structural variants detected via karyotyping.

In conclusion, our study highlights the critical role of WGS in resolving challenging NDDs cases with *de novo* structural rearrangements. However, despite its diagnostic power, WGS remains cost-prohibitive and computationally demanding, limiting its widespread implementation in routine clinical settings. Consequently, many patients with atypical or less defined phenotypes may harbor undetected balanced rearrangements or cryptic SVs that elude standard genetic testing. The advent of long-read sequencing technologies promises to overcome these limitations by enhancing the resolution of complex SVs, repeat expansions, and epigenetic modifications, which are frequently missed by short-read sequencing approaches. As sequencing costs decline and bioinformatics tools for SVs interpretation continue to evolve, the routine clinical adoption of WGS is poised to revolutionize genetic diagnostics, enabling earlier and more precise identification of pathogenic variants in patients with complex genetic disorders.

## Data Availability

The original contributions presented in the study are included in the article/Supplementary Material; further inquiries can be directed to the corresponding author.
