# Supplementary material for: Integrating genome and transcriptome analysis to decipher balanced structural variants in unsolved cases of neurodevelopmental disorders
Source: Front Genet. 2025 Jul 7;16:1603513. doi: 10.3389/fgene.2025.1603513 (PMC12277603; doi:10.3389/fgene.2025.1603513)
Supplement: Supplementary file 1 [file Table1.docx]

Table S1: Primers used for sequencing breakpoint regions and fusion transcripts, along with corresponding PCR product lengths.

| ***CHD7* gene breakpoint's primers** | | |
| --- | --- | --- |
| SLC20A2_IVS3F: | 5'-tgaaaatgccccagatggtt-3' | **487 bp** in case of Chromosomic Inversion |
| CHD7_IVS4R: | 5'-actttgagtgccagatgtca-3' |  |
|  | |  |
| ***SLC20A2* gene breakpoint's primers** | | |
| CHD7_IVS4F: | 5'-cgagccttggttatttggcat-3' | **323 bp** in case of Chromosomic Inversion |
| SLC20A2_IVS3R: | 5'-aagccctgtgacttggcaga-3' |  |
|  | |  |
| ***SLC20A2-CHD7*** **fusion transcript's primers** | | |
| SLC20A2_ex3F: | 5'-CCGCTGTGTGGCAGCTGATT-3' | PCR product sizes corresponding to exon 4 inclusion or skipping events: **271 bp** when exon 4 is skipped in both *SLC20A2* and *CHD7*;  **357 bp** when exon 4 is skipped only in *CHD7*;  **413 bp** when exon 4 is skipped only in *SLC20A2* and **499 bp** when exon 4 is retained in both genes. |
| CHD7_ex5R: | 5'-GTTCTGACTGGGAGGTGTTGG-3' |  |
|  | |  |
| ***CHD7-SLC20A2* fusion transcript's primers** | | |
| SLC20A2_ex5F: | 5'-CCCTGTTCCCAATGGCCTCC-3' | PCR product sizes corresponding to exon 4 inclusion or skipping events: **512 bp** when exon 4 is skipped in both *SLC20A2* and *CHD7*;  **598 bp** when exon 4 is skipped only in *CHD7*;  **654 bp** when exon 4 is skipped only in *SLC20A2* and **740 bp** when exon 4 is retained in both genes. |
| CHD7_ex3R: | 5'-GCTGGATTTGGGTTTTGGCG-3' |  |
