# Supplementary figures and images for: Integrating genome and transcriptome analysis to decipher balanced structural variants in unsolved cases of neurodevelopmental disorders
Source: Front Genet. 2025 Jul 7;16:1603513. doi: 10.3389/fgene.2025.1603513 (PMC12277603; doi:10.3389/fgene.2025.1603513)

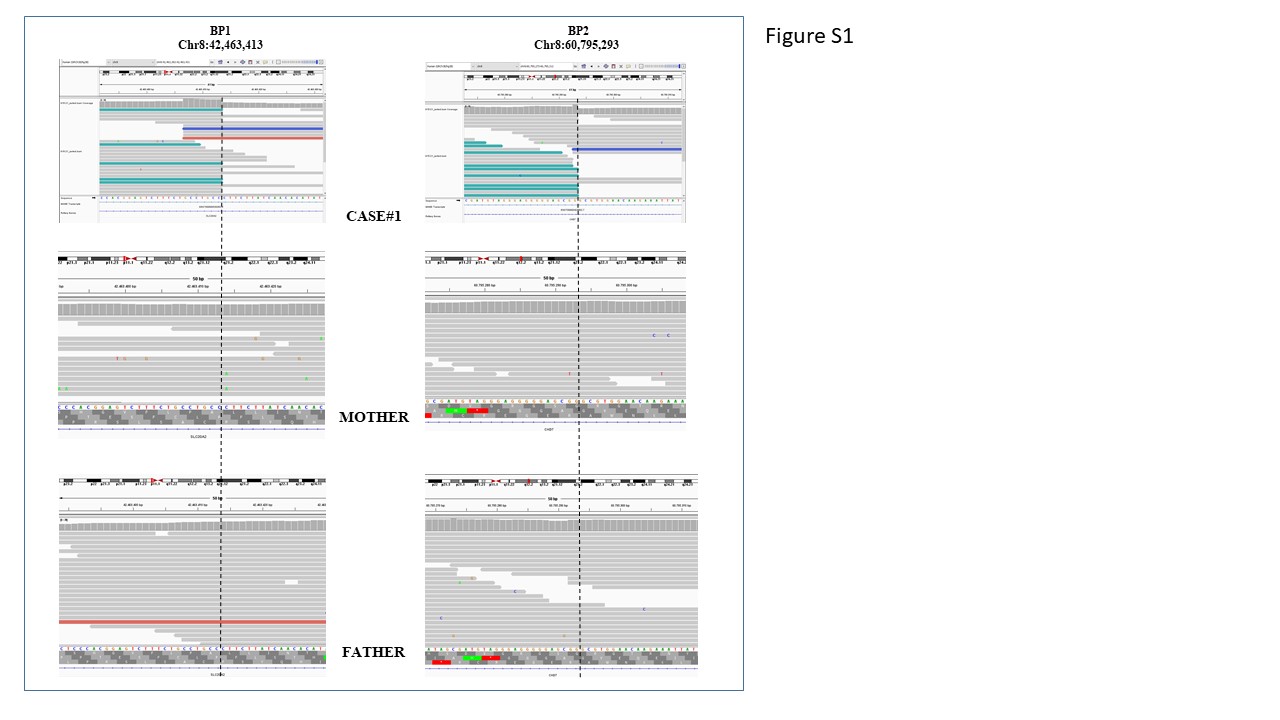

Supplement: Supplementary file 2 [file Image1.jpeg]

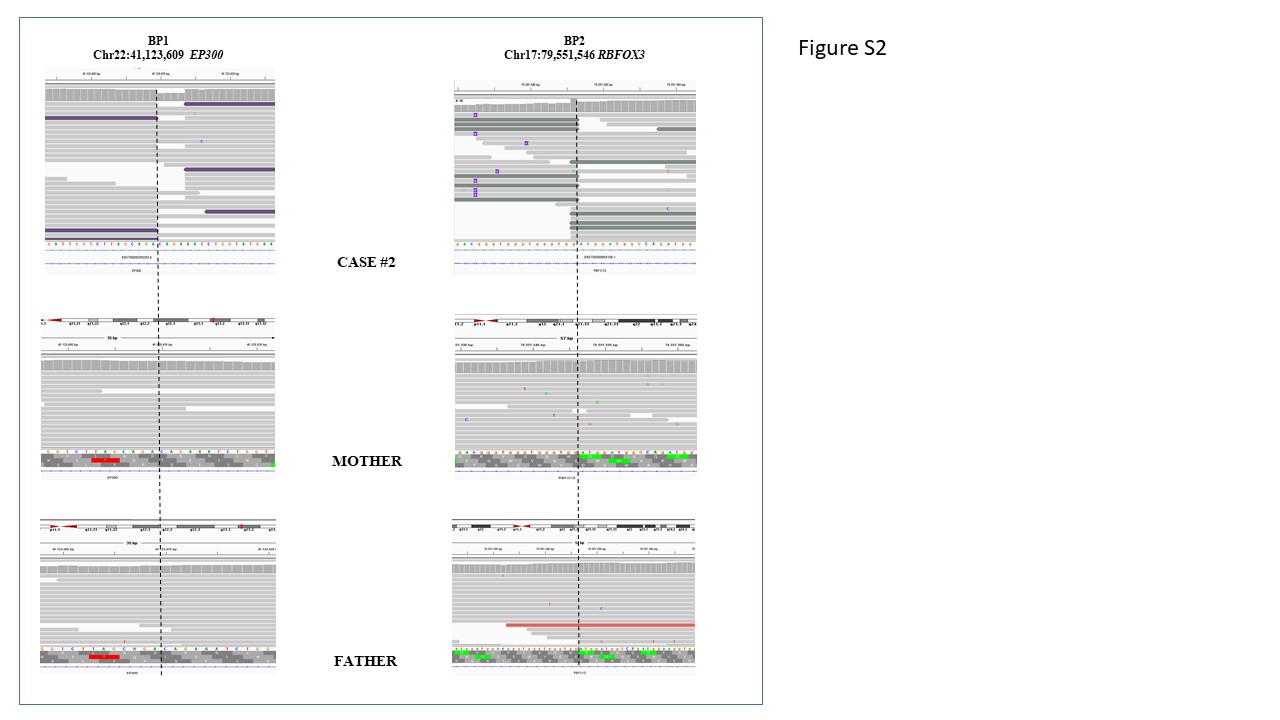

Supplement: Supplementary file 3 [file Image2.jpeg]
